# Staged Endodontic Management and Esthetic Rehabilitation of a Fractured Immature Incisor With Failed Endodontic Treatment and Iatrogenic Complications: A Case Report

**DOI:** 10.1002/ccr3.73893

**Published:** 2026-11-06

**Authors:** Prerna Priya, Abiskar Basnet

**Affiliations:** ^1^ Department of Dentistry AIIMS Rishikesh Uttarakhand India; ^2^ Department of Dentistry ESIC Medical College and Hospital Faridabad Haryana India; ^3^ Department of Dentistry and Maxillofacial Surgery Birat Medical College Teaching, Hospital Biratnagar Nepal

**Keywords:** biodentine, endodontic retreatment, endodontic surgery, fiber‐reinforced composite, open apex, platelet‐rich fibrin

## Abstract

Managing a previously treated immature incisor with multiple concurrent structural, endodontic, and periapical complications is clinically challenging. This report describes staged surgical, nonsurgical endodontic, and restorative management of a fractured, discolored tooth with a blunderbuss canal, overextended gutta‐percha, apicomarginal defect, cervical perforation, and periapical cyst.

## Key Clinical Message

A conservative, staged decision‐making approach can effectively facilitate preservation of a compromised immature incisor, even when multiple procedural and structural complications coexist by prioritizing multidisciplinary treatment over extraction.

## 1 Introduction

The management of endodontically failed immature permanent anterior teeth poses significant endodontic and restorative challenges. Pulp necrosis arrests root development, leaving an open apex, thin dentinal walls, and an unfavorable crown–root ratio. These factors contribute to endodontic failures, increase fracture risk, and may compromise tooth retention [1].

Apexification induces the formation of a mineralized apical barrier in nonvital immature teeth with open‐apices. Calcium hydroxide apexification may weaken dentin, provide poor apical seal, and require prolonged treatment, repeated follow‐up, and sustained patient compliance [2, 3, 4]. Mineral trioxide aggregate (MTA) and Biodentine apical plugs have increasingly replaced calcium hydroxide apexification because they have excellent sealing ability and eliminate the need to wait for the formation of a hard‐tissue apical barrier [2, 4].

Regenerative endodontic procedures (REPs) have limitations in teeth with extensive structural loss because, without the placement of a fiber‐post and/or a bonded restoration, the remaining tooth structure may not withstand functional and parafunctional forces [1, 2].

A blunderbuss‐canal further complicates apical control and the establishment of an adequate seal [5]. Therefore, the apical matrix technique was developed to provide a barrier against which MTA or Biodentine could be condensed [2].

Platelet‐rich fibrin (PRF), an autologous second‐generation platelet concentrate, may serve as a resorbable matrix material. PRF provides a bioactive scaffold rich in growth factors, including PDGF, TGF‐β, and VEGF, thereby promoting tissue healing and cellular regeneration [2]. Previous studies have successfully used PRF membranes as apical barriers for improved apical seal, predictable periapical healing, and controlled placement of MTA or Biodentine [2, 5, 6].

Iatrogenic errors and inadequate apical or coronal seals may also contribute to endodontic failure [5]. Clinicians should confine endodontic materials within the root canal because their extrusion into the periapical tissues may cause pain or tissue necrosis.

The loss of cervical tooth structure affects the biomechanical properties of the tooth and increases the risk of tooth fracture [7]. Bidirectional glass fiber mesh may improve stress distribution and reinforce structurally compromised immature anterior teeth after endodontic treatment [8].

Apicomarginal defects, characterized by complete localized loss of marginal bone that communicates with a periodontal pocket, pose a greater healing challenge than defects confined to the periapical area [9]. Conservative endodontic surgery removes periapical pathology and promotes healing. Adequate disinfection and sealing may permit lesion management without root‐end resection, thereby preserving tooth structure and reducing postoperative complications [10].

Intracoronal bleaching treats discolored teeth. Carbamide peroxide, sodium perborate, and hydrogen peroxide effectively bleach discolored teeth and are less invasive than crowns [11].

The concurrence of these endodontic, periodontal, restorative, and esthetic complications further complicated retreatment. Although the individual procedures used in this case are established, limited evidence exists regarding their staged application in a fractured, discolored, previously treated immature incisor presenting concurrently with blunderbuss‐canal, overextended root‐filling material, apicomarginal defect, cervical perforation, and a histologically confirmed periapical cyst. This report describes the clinical reasoning and sequential surgical, nonsurgical endodontic, and restorative management used to preserve the affected tooth over an 18‐month follow‐up period.

## 2 Case History/Examination

The present case report follows the CAse REport (CARE) guidelines. A 19‐year‐old male patient with no relevant medical history presented to the Department of Dentistry with the chief complaint of a discolored, fractured maxillary anterior tooth and mild‐to‐moderate continuous pain during mastication. He reported childhood dental trauma followed by root canal treatment approximately 10 years earlier.

Clinical examination showed discoloration, a dislodged restoration, an Ellis Class IV fracture, and a non‐draining sinus tract associated with the permanent maxillary left central incisor (tooth #9) (Figure 1A). The tooth showed no mobility and responded positively to both percussion and palpation and had a buccal periodontal probing depth of 6 mm. Teeth #7, #8, and #10 responded positively to both cold and electric pulp testing, whereas tooth #9 showed no response to either test.

**FIGURE 1:**
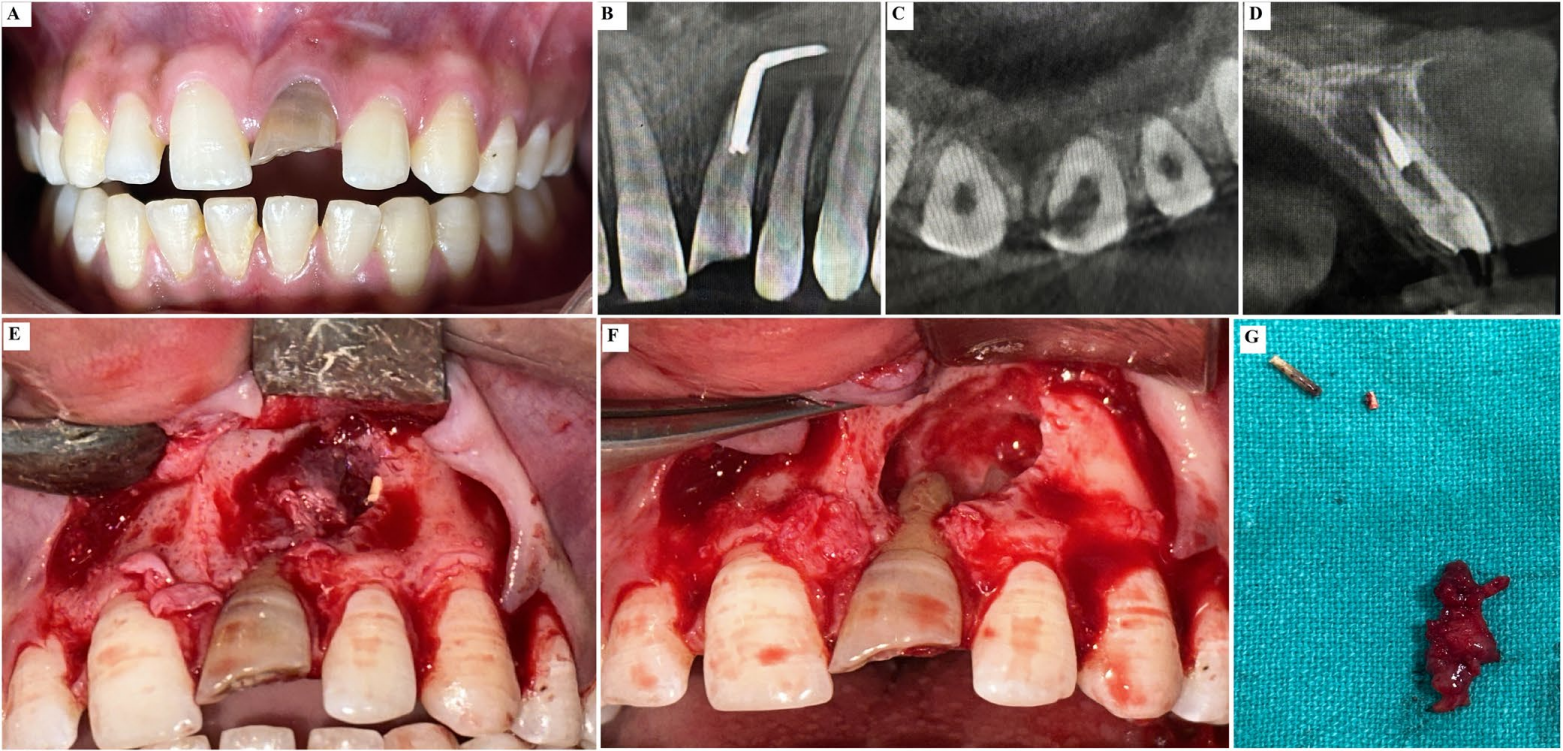
(A) Preoperative clinical image. (B–D) Preoperative coronal, axial, and sagittal cone‐beam computed tomographic views, respectively. (E) Surgical exposure showing granulation tissue and extruded gutta‐percha. (F) Apicomarginal defect involving tooth #9. (G) Excised granulation tissue and surgically retrieved gutta‐percha.

## 3 Methods (Investigations, Differential Diagnosis, and Treatment)

A periapical radiograph revealed an endodontically treated immature tooth with a blunderbuss‐canal and thin dentinal walls, markedly overextended gutta‐percha in relation to tooth #9 and a large, well‐defined periapical radiolucency involving teeth #9 and #10. Limited‐field‐of‐view (LFOV) CBCT imaging delineated the lesion and the extent of gutta‐percha extrusion. CBCT revealed the following findings (Figure 1B–D):

1. A well‐defined periapical lesion measuring 2.0 cm × 1.8 cm, extending palatally and superiorly toward the nasal floor.
2. Iatrogenic cervical perforation on the mesial aspect of tooth #9.
3. A 9‐mm long L‐shaped extrusion of gutta‐percha beyond the apex.

Based on the clinical and radiographic findings, a pulpal diagnosis was of a previously treated and a periapical diagnosis was of a chronic apical abscess (American Association of Endodontists [AAE] endodontic diagnosis).

The treatment plan included nonsurgical or surgical retrieval of the overextended gutta‐percha, PRF matrix‐assisted Biodentine apexification and perforation repair, intracoronal bleaching, and esthetic rehabilitation with fiber‐reinforced composite. The proposed treatment, alternative options, and prognosis were explained to the patient, and written informed consent for treatment was obtained. All procedures were performed under 5.0× magnification (Admetec, Haifa, Israel) and rubber‐dam isolation.

Nonsurgical retrieval of the extruded gutta‐percha was attempted first. However, its L‐shaped configuration and extension into the periapical lesion prevented retrieval with hand files. Surgical retrieval was therefore undertaken. One week later, the canal was debrided and irrigated, and intracanal medication was placed.

A presurgical rinse with 0.2% chlorhexidine was performed. Following local anesthesia with 2% lidocaine containing 1:100,000 epinephrine, a full‐thickness mucoperiosteal flap was reflected (Figure 1E). The lesion was carefully curetted, and the extruded gutta‐percha was removed. An apicomarginal defect involving tooth #9 was evident (Figure 1F,G). No apicoectomy was performed, and no regenerative materials were used. The flap was repositioned and sutured. Standard postoperative instructions were given. The sutures were removed after 7 days, and healing was uneventful. Histopathological examination of the excised lesion confirmed a periapical cyst. However, a detailed microscopic description and photomicrographs were not available for inclusion in this report.

The patient was recalled after 1 week. The working length was established 2 mm short of the radiographic root length using an electronic apex locator (Root ZX Apex Locator, J. Morita Corporation, Kyoto, Japan) and a periapical radiograph. The canal was gently debrided with *K*‐files (Dentsply Maillefer, Ballaigues, Switzerland) using circumferential filing to disrupt the bacterial biofilm and preserve the thin root walls. The canal was irrigated with 2% sodium hypochlorite and EDTA using a side‐vented needle. A final flush using saline was performed. The canal was then dried with sterile paper points. Triple antibiotic paste comprising ciprofloxacin, metronidazole, and minocycline (1:1:1), each at 100 μg/mL, was mixed with propylene glycol and placed 1 mm short of the radiographic apex. A temporary restoration of resin‐modified glass ionomer cement (Fuji II LC, GC America) was placed. The patient was recalled after 2 weeks.

At the subsequent appointment, the temporary restoration and intracanal medicament were removed, and the canal was irrigated and dried. PRF was prepared following the protocol described by Choukroun et al. [12] 10 mL of the patient's venous blood was collected in sterile tubes without anticoagulant. The blood samples were centrifuged at 3000 rpm for 10 min in a tabletop centrifuge (REMI R8C, Mumbai, India), producing an upper acellular plasma layer, a central PRF clot, and a lower red blood cell layer (Figure 2A). The PRF clot was gently compressed between sterile gauze to form a membrane. The membrane was then placed into the canal and gently condensed with a preselected master cone to create a stable apical matrix.

**FIGURE 2:**
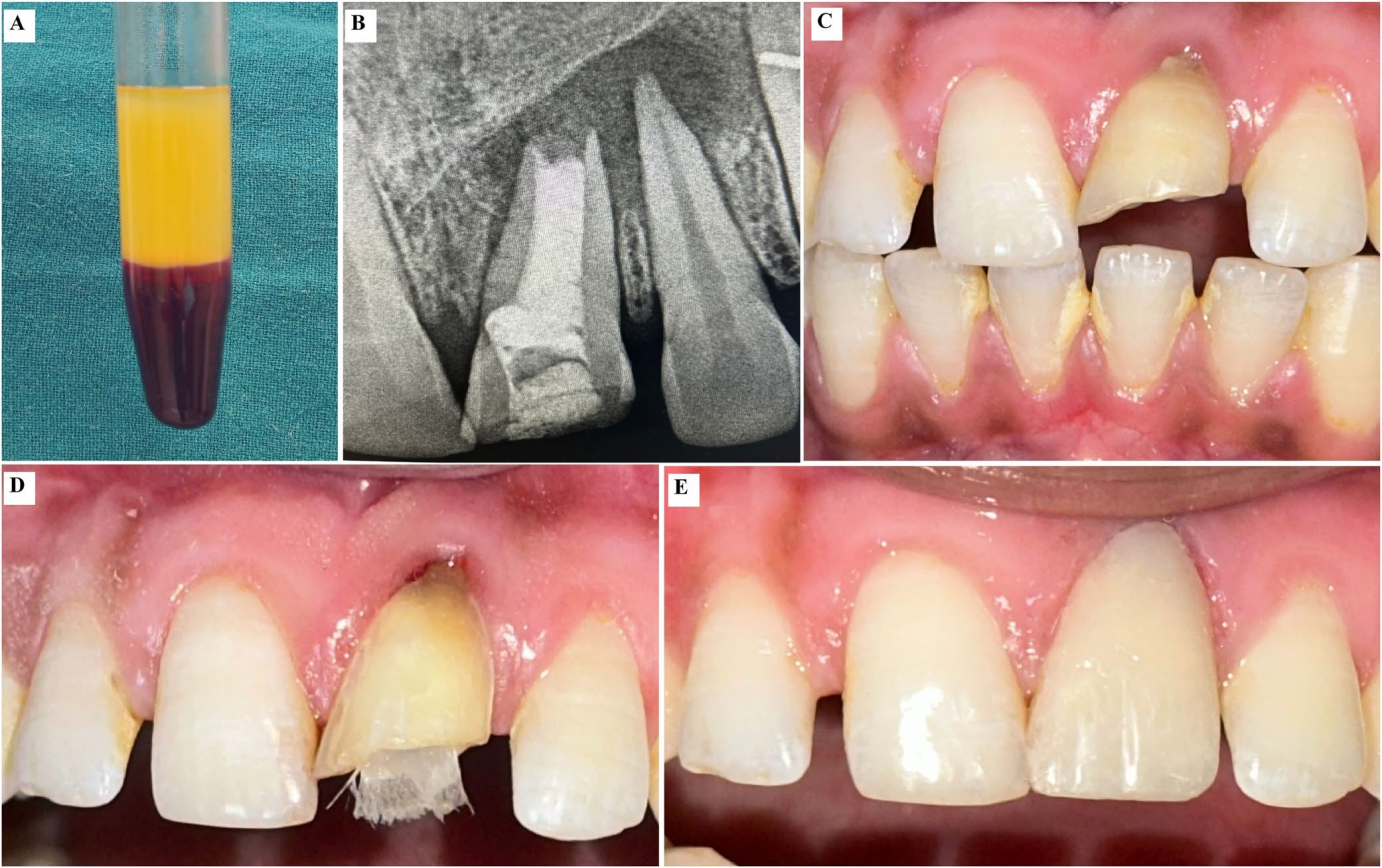
(A) Test tube containing platelet‐rich fibrin after centrifugation. (B) Immediate postoperative periapical radiograph showing cervical perforation repair and Biodentine obturation. (C) Clinical image recorded after four cycles of intracoronal bleaching; Cairo gingival recession type 1 (RT1) and resolution of the sinus tract were noted. (D) Clinical view after tooth preparation and placement of a bidirectional glass fiber mesh within the coronal canal space. (E) Immediate postoperative labial view after esthetic rehabilitation.

Biodentine (Septodont, Saint‐Maur‐des‐Fosses, France) was prepared as recommended by the manufacturer and placed over the PRF matrix. It was condensed with hand pluggers. The remaining root‐canal was obturated with Biodentine, and the cervical perforation was repaired from the intracanal aspect with the same material. A postoperative radiograph confirmed its placement (Figure 2B).

At the next visit, 2 days later, intracoronal bleaching using 30% hydrogen peroxide and sodium perborate (Degussa, Hanau, Germany) was performed to correct the discoloration. A cotton pellet coated with dentin‐bonding agent was positioned over the bleaching material and light‐cured before placement of an adhesive temporary restoration (Filtek Z350, 3 M ESPE, MN, USA). The bleaching procedure was repeated at 7‐day intervals. A mild shade improvement was noted after 4 visits. Follow‐up radiographs were taken to rule out external cervical resorption. The bleaching agent was removed, and the pulp chamber was thoroughly irrigated. A cotton pellet soaked in catalase was placed for 3 min, followed by a final rinse.

Cairo gingival recession Type 1 (RT1) developed 8 weeks after the endodontic surgery [13] (Figure 2C). A root‐coverage procedure was offered, but the patient declined.

Because a shade mismatch with the adjacent teeth remained, a direct composite veneer and reinforcement of the canal with a preimpregnated glass fiber strip were planned. The patient declined rubber dam isolation during the restorative phase. Moisture control was therefore maintained using cotton rolls, a saliva ejector, and high‐volume suction.

After tooth preparation, a Dentapreg UFM (ADM A.S., Brno, Czech Republic) strip was cut and adapted within the coronal canal space (Figure 2D). The tooth surface was etched, rinsed, dried, and treated with an adhesive system. A thin layer of uncured composite resin was applied, and the fiber strip was embedded and adapted within it. The strip was light‐cured and completely covered with composite resin (Filtek Z350 XT, 3 M ESPE, MN, USA). The restoration was built incrementally. It was then finished, polished, and adjusted for occlusion (Figure 2E).

## 4 Conclusions and Results (Outcome and Follow‐Up)

Clinical and radiographic examinations at 3, 6, 12, and 18 months showed that the tooth remained asymptomatic at all follow‐up visits, with resolution of the sinus tract, no tenderness to percussion or palpation, and no mobility (Figures 3A–D and 4). At 18 months, the probing depth was reduced from 6 mm to 2 mm, and the recessed gingival margin remained stable. Radiographic healing was assessed qualitatively by comparing pretreatment and follow‐up periapical radiographs; no quantitative measurement of lesion reduction was performed. Radiographic evaluation demonstrated progressive osseous healing and a substantial reduction in the periapical radiolucency, although a small residual apical radiolucency and absence of further root development remained at 18 months. However, the development of gingival recession highlights the need for careful periodontal assessment and long‐term follow‐up in cases involving extensive osseous defects. Following an 18 month follow‐up examination, the patient was asymptomatic and highly satisfied that the tooth has been preserved, as well as with its appearance and function.

**FIGURE 3:**
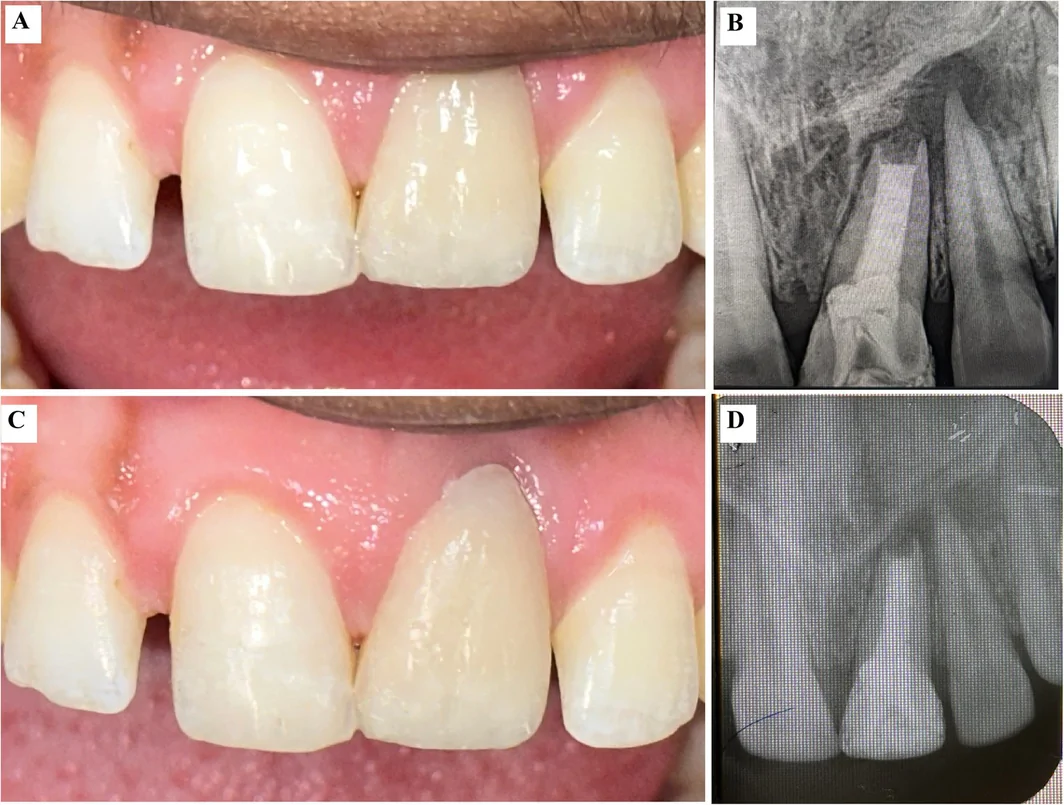
(A) Clinical image at the 12‐month follow‐up. (B) Periapical radiograph at the 12‐month follow‐up. (C) Clinical image at the 18‐month follow‐up showing a stable gingival margin. (D) Periapical radiograph at the 18‐month follow‐up showing a substantial reduction in the periapical radiolucency.

**FIGURE 4:**
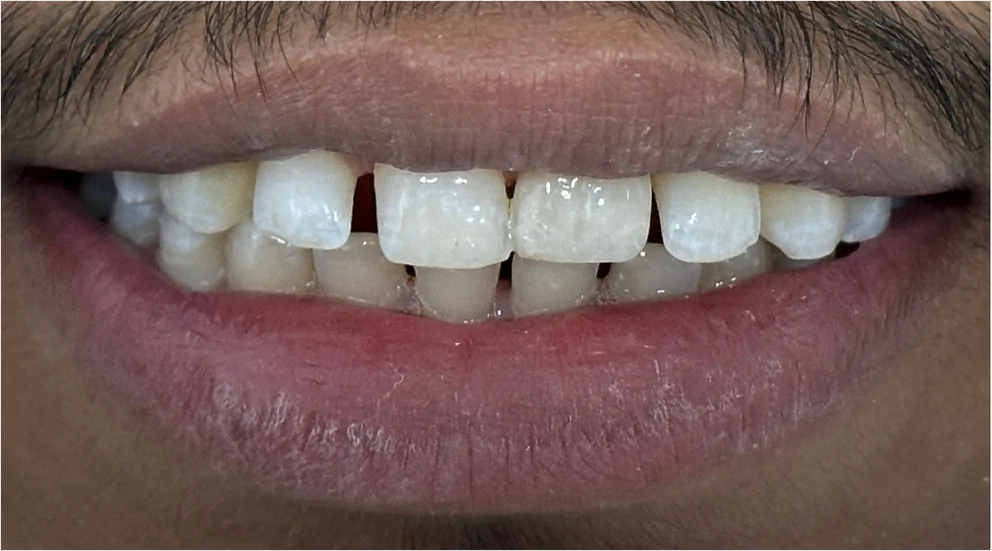
Frontal smile view at the 18‐month follow‐up.

Staged endodontic and restorative management retained the fractured, previously treated immature incisor with a large periapical radiolucency. The combined use of magnification and CBCT‐assessment facilitated precise management while preserving the remaining tooth structure.

## 5 Discussion

Conventional periapical radiographs provide only a two‐dimensional image and may fail to detect perforations or the full extent of the periapical lesion. In this case, LFOV CBCT provided a three‐dimensional assessment of the tooth and periapical defects for diagnosis and treatment planning [3].

The thin dentinal walls necessitated minimal instrumentation to preserve the remaining root structure, with intracanal medication serving as an adjunct to canal disinfection. Triple antibiotic paste (TAP) was used as the intracanal medicament to enhance disinfection of the previously treated canal because calcium hydroxide may have limited activity against microorganisms associated with secondary endodontic infections, including *Enterococcus faecalis* and *Candida species* [5]. Its antimicrobial activity may be partly attributed to the ability of the combined antibiotics to penetrate the dentinal tubules. Its use also minimizes postoperative pain. In the present case, TAP was used at a low concentration of 100 μg/mL. This concentration was selected in view of reports indicating no detectable adverse effects on stem cells from the apical papilla (SCAP) at 0.1 and 0.01 mg/mL, whereas concentrations above 1 mg/mL may compromise cell survival [14].

In the present case, apicoectomy and a full‐coverage crown were avoided to prevent further weakening of the already compromised tooth structure. In teeth with pre‐existing periodontal destruction and marginal bone loss, root‐end resection may further compromise the root‐to‐crown ratio (RCR) and remaining periodontal support [15]. Although apicoectomy is commonly performed during periapical surgery, root‐end resection may be avoided in selected cases when adequate canal disinfection and sealing can be achieved. Preserving the root apex conserves the strength and stability of the tooth and may reduce complications associated with resection [10, 16].

Previous studies have advocated guided tissue regeneration (GTR) for apicomarginal defects to limit epithelial downgrowth and postoperative gingival recession. However, a randomized controlled trial reported comparable healing outcomes in apicomarginal defects treated with and without PRF [9]. In the present case, no GTR material was placed over the defect; surgical debridement and subsequent intracanal disinfection were prioritized, allowing healing from the adjacent periodontal ligament [9].

In the present case, PRF was used as an internal apical matrix to support controlled placement of Biodentine. The cytokines present in PRF are gradually released as the fibrin matrix disintegrates over 7–11 days. PRF may promote angiogenesis, fibrin remodeling, and cellular proliferation. Its autologous origin and biocompatibility make PRF a suitable apical matrix for teeth with wide‐open apices [5]. Direct placement of Biodentine at an open apex may potentially affect continued root development because its high alkalinity (pH≈12.5) can adversely influence cells of Hertwig's epithelial root sheath (HERS) and the apical papilla [4].

In the present case, Biodentine served multiple purposes during the staged management. It functioned as an apical barrier, an obturation material, a perforation‐repair material, and a cervical barrier during bleaching. One study reported that Biodentine may reinforce immature roots and restore tooth function when used as an obturating material. Its compressive strength reaches a value comparable to that of natural dentin [17]. It demonstrates superior physical properties and biocompatibility compared with MTA, possibly because greater hydroxyapatite deposition on its surface was seen after being exposed to tissue fluids [18].

A bidirectional E‐glass fiber network was incorporated within the composite restoration in the present case. A fiber post was avoided because additional space preparation could have further weakened the thin root walls. Multidirectional or woven fibers may perform better than unidirectional fibers because they may enable restorations to withstand forces from multiple directions during mastication [19].

Internal bleaching was undertaken after completion of the endodontic and surgical phases. An adhesive temporary restoration minimizes recurrent discoloration and dentinal recontamination between appointments. A cervical barrier reduces the risk of external cervical resorption. In this case, Biodentine also served as the cervical barrier as it provides a better seal than glass ionomer cement. Catalase neutralized residual oxidants and facilitated immediate placement of the definitive adhesive composite restoration [20].

At the 18‐month follow‐up, the periapical radiolucency had substantially reduced, with a small residual radiolucent area. However, root maturation was not observed. HERS contributes to continued root elongation, thickening of the dentinal walls, formation of cementum, and eventual apical closure [4]. Periapical inflammation releases TNF‐α and IL‐1, which impair the function of HERS and stem cells from the apical papilla (SCAP) and disrupt normal root maturation. Controlling infection and inflammation within the pulp–periapical tissues is essential to create conditions that may allow surviving apical papilla cells and HERS to regain function, potentially enabling root development to resume in immature permanent teeth with pulp necrosis and apical periodontitis [17]. In this case, the absence of continued root maturation was more plausibly associated with longstanding periapical inflammation. The condition of HERS and the apical papilla was not assessed; therefore, the absence of further root development cannot be attributed specifically to injury of these tissues. However, a previous case series similarly reported absent root development, possibly because of severe HERS damage [4].

Cairo Type 1 (RT1) gingival recession developed 8 weeks after endodontic surgery and represented an important postoperative complication in this case. This complication emphasizes the importance of discussing potential periodontal and esthetic consequences before treatment and evaluating gingival outcomes alongside periapical healing and tooth retention. The patient's presurgical and postsurgical oral hygiene, full‐thickness flap elevation, periodontal phenotype, papillary morphology, lesion dimensions, and individual healing capacity may affect the postoperative position of the gingival margin. Complete mobilization of the interdental papilla, which can occur due to full‐thickness flap elevation, may disrupt its vascular supply, increasing the risk of tissue injury and necrosis. Recession may also be associated with inadequate adaptation of the papilla to the underlying bone during flap repositioning and excessive mechanical force during flap reflection [21]. Multiple factors may have contributed, including the preexisting apicomarginal defect. Gingival recession may expose the root surface, increasing susceptibility to non‐carious cervical lesion, dentinal hypersensitivity, and root caries, while also adversely affecting both function and aesthetics. A root‐coverage procedure was therefore offered, but the patient declined further surgery. Supportive periodontal care, plaque control, and maintenance of a smooth restorative surface were therefore emphasized. A systematic review reported that deep margin elevation using composite is well‐tolerated by the periodontal tissue if the composite is well‐polished, maintained biological width integrity, strict supportive therapy, and good oral hygiene practices [22].

The absence of rubber dam isolation during the adhesive restorative procedure was the main limitation, as inadequate moisture control may reduce the predictability of adhesive bonding. Follow‐up images were limited to periapical radiographs due to the patient's financial constraints. Although these images demonstrated progressive reduction of the radiolucency, they did not permit three‐dimensional assessment of the residual lesion or cortical bone repair. Complete three‐dimensional osseous healing could therefore not be confirmed. As a single case report, the findings cannot be generalized to other immature teeth with comparable iatrogenic complications.

When overextended filling material and a large periapical lesion coexist in a previously treated immature tooth, orthograde retreatment alone may not address all causes of failure. A staged surgical–orthograde strategy can preserve root length and permit tooth retention, although periodontal outcomes require continued monitoring. This case may assist clinicians in selecting conservative multidisciplinary treatment instead of extraction when several procedural and structural complications coexist.

## Author Contributions

**Prerna Priya:** conceptualization, methodology, investigation, data curation, project administration, resources, validation, visualization, writing – original draft, writing – review and editing. **Abiskar Basnet:** conceptualization, methodology, investigation, resources, validation, writing – review and editing.

## Funding

The authors have nothing to report.

## Ethics Statement

The authors have nothing to report.

## Consent

According to the institutional policy of AIIMS Rishikesh, where this case was managed, formal approval from the Institutional Ethics Committee was not required for the publication of an individual case report. Nevertheless, written informed consent was obtained from the patient presented in the case report for treatment and publication.

## Conflicts of Interest

The authors declare no conflicts of interest.

## Data Availability

The data that support the findings of this study are available from the corresponding author upon reasonable request.
